# Supplementary material for: De novo origins of multicellularity in response to predation
Source: Sci Rep. 2019 Feb 20;9:2328. doi: 10.1038/s41598-019-39558-8 (PMC6382799; doi:10.1038/s41598-019-39558-8)
Supplement: Supplementary file 10 — Supplementary Material [file 41598_2019_39558_MOESM10_ESM.doc]

// This macro was designed to count nuclei per cluster.

// It will prompt you to select a directory and analyze all files it finds there

// that end in "b.jpg" (incl subfolders). See line 52 to modify the filename

// filter for your own uses.

//

// PS: For each file it should produce a .csv with results, a .zip file with ROI

// data, and a .txt with the log data.

//

// This script goes heavy on the logging. However, the threshold values are logged,

// too, so you can go back and see what they were. Useful, I hope.

//

// -JSW, last updated 6 Apr 2016

requires("1.47g");

dir = getDirectory("Choose a Directory: ");

setBatchMode(false);

count = 0;

countFiles(dir);

n = 0;

processFiles(dir);

//print(count+" files processed");

run("Input/Output...", "file=.csv");

print("\\Clear"); //this empties the log

function countFiles(dir) {

list = getFileList(dir);

for (i=0; i<list.length; i++) {

if (endsWith(list[i], "/"))

countFiles(""+dir+list[i]);

else

count++;

}

}

function processFiles(dir) {

list = getFileList(dir);

for (i=0; i<list.length; i++) {

if (endsWith(list[i], "/"))

processFiles(""+dir+list[i]);

else {

showProgress(n++, count);

path = dir+list[i];

processFile(path);

}

}

}

function processFile(path) {

if (endsWith(path, "b.jpg")) {

open(path);

name = getTitle;

index = lastIndexOf(name,".");

if (index!=-1)

name = substring(name,0,index);

name1 = name + ".csv";

name2 = name + ".zip";

name3 = name + ".txt";

IJ.log("Opened " + path + "...");

run("8-bit");

IJ.log("Converted to 8-bit. Now thresholding...");

run("Auto Threshold", "method=Triangle white setthreshold show");

//setThreshold(10, 255);

run("Convert to Mask");

IJ.log("Converted to mask. Now filling holes and dilating...");

run("Fill Holes");

run("Dilate");

run("Dilate");

IJ.log("Done. Now analyzing particles...");

run("Analyze Particles...", "size=50-Infinity display clear summarize add in_situ");

IJ.log("Time to wait for half a minute! Doot doot doot...");

wait(30000);

IJ.log("OK, done waiting. Now reverting.")

run("Clear Results");

run("Revert");

run("Find Maxima...", "noise=7 output=[Single Points] exclude");

IJ.log("Cool? Cool. Closing all other image windows and running ROI manager...");

close("\\Others"); //closes all but the active window

run("ROI Manager...");

roiManager("Show None");

roiManager("Show All");

run("Set Measurements...", "area mean min integrated redirect=None decimal=3");

IJ.log("Measuring...");

roiManager("Measure");

wait(30000);

IJ.log("Done. Saving results to file(s).");

roiManager("Save", dir+name2);

saveAs("Results", dir+name1);

selectWindow("Log");

saveAs("Text", dir+name3);

print("\\Clear");

close();

}

}
